# Pre-emptive Intracoronal Radiolucency in First Permanent Molar

**DOI:** 10.5005/jp-journals-10005-1502

**Published:** 2018-04-01

**Authors:** Mariana C Ilha, Paulo F Kramer, Simone H Ferreira, Henrique C Ruschel

**Affiliations:** 1 PhD Student, Department of Pediatric Dentistry, Lutheran University of Brazil (ULBRA), Canoas, RS, Brazil; 2 Professor, Department of Pediatric Dentistry, Lutheran University of Brazil (ULBRA), Canoas, RS, Brazil; 3 Professor, Department of Pediatric Dentistry, Lutheran University of Brazil (ULBRA), Canoas, RS, Brazil; 4 Professor, Department of Pediatric Dentistry, Lutheran University of Brazil (ULBRA), Canoas, RS, Brazil

**Keywords:** Dentin, Dentition permanent, Tooth crown, Tooth resorption.

## Abstract

Pre-eruptive intracoronal radiolucency (PECR) is characterized by the existence of a radiographic radiolucent area inside the coronal dentin prior to dental eruption. It is a rare clinical entity of unknown etiology, usually asymptomatic and diagnosed through routine radiographs. The aim of this article is to report the treatment of a PECR in an 8-year-old girl. Surgical procedure to expose the crown was conducted; upon tooth eruption, the radiolucent lesion was accessed and the tooth was restored. The case has an 18-month follow-up and emphasis on aspects linked to diagnosis and clinical approach was discussed.

**How to cite this article:** Ilha MC, Kramer PF, Ferreira SH, Ruschel HC. Pre-eruptive Intracoronal Radiolucency in First Permanent Molar. Int J Clin Pediatr Dent 2018;11(2):151-154.

## INTRODUCTION

Pre-eruptive intracoronal radiolucency is a dental anomaly characterized by the existence of a radiographic radiolucent area inside the coronal dentin of a dental element prior to its eruption. Other terms used to describe the clinical condition are pre-eruptive dentin radiolu-cency, pre-eruptive coronal resorption, pre-eruptive intracoronal resorption, and pre-eruptive caries.^1,2^

The etiology of this anomaly remains unknown. It is similar to the process of occult caries, but differs from the latter because the tooth has no communication with the oral cavity, such that cariogenic bacteria are unlikely to reach the enamel.^3,4^ The PECR is a rare condition, mainly associated with mandibular permanent molars and premolars.^1,2^

Lesions are usually located at the central or mesial portion of the coronary dentin, close to the ameloden-tinal junction; depending on their evolution, lesions may reach the pulp tissue.^3,5,6^ The diagnosis is made through routine radiographs or radiographs taken for orthodontic purposes. Early diagnosis is extremely important due to the risk of pulp damage on the affected dental element.^2^ Thus, restorative intervention is necessary.

The aim of this article is to report a case of PECR in the left mandibular first permanent molar of a young female patient, with emphasis on aspects linked to diagnosis and clinical approach.

## CASE REPORT

**Fig. 1:**
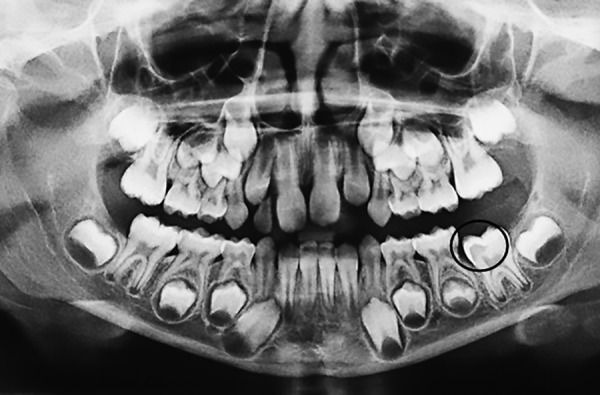
Panoramic radiograph showing a radiolucent area on the coronal portion of the left mandibular first permanent molar of the patient

The patient, an 8-year-old girl, came to the Lutheran University of Brazil for a routine dental consultation. Upon clinical examination, it was observed that the patient presented chronology (dentition development) and sequence of eruption compatible with her age; however, the left mandibular first permanent molar was absent. No color, texture, or volume changes on the mucosa were observed; the patient did not experience pain. The panoramic radiograph (Fig. 1) showed an alteration on the nonerupted first permanent molar, represented by a radiolucent dentin area at the occlusal surface, more like mesial. Based on both clinical and radiographic aspects, the lesion was diagnosed as PECR.

**Figs 2A and B:**
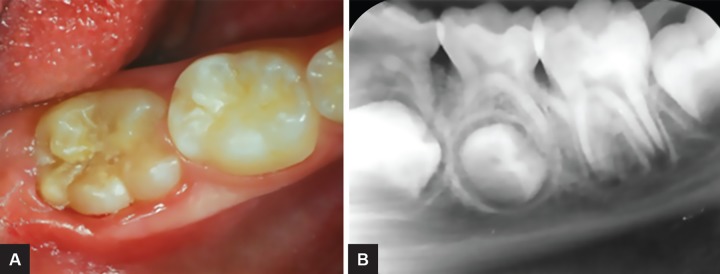
(A) Left mandibular first permanent molar partially erupted 6 months after surgical intervention. (B) Radiograph image of the first permanent molar 6 months after surgical intervention

**Figs 3A and B:**
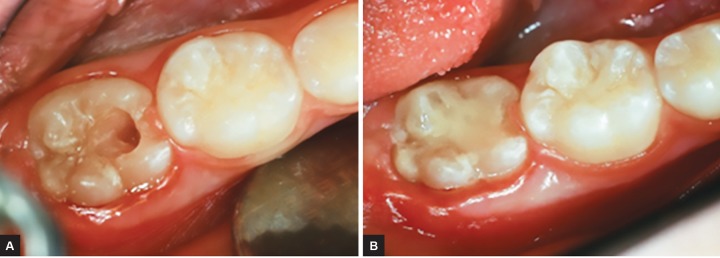
(A) Clinical access to the radiolucent area shown on the radiograph. (B) Restoration of the area compromised by radiolucent lesion, made with glass ionomer

The treatment was conducted in two parts; first, the crown was surgically exposed by removing the gingival mucosa that covered the unerupted tooth. This procedure allowed access to the occlusal surface of the tooth, which was sound.

After 6 months, during the second phase of the treatment, the first molar was partially erupted (Fig. 2). Clinically, there were no structural or color changes on the crown. After local anesthesia, the site corresponding to the radiolucent area was reached; enamel and dentin that covered the lesion were removed using rotating instruments, until the affected area was reached, where an “empty space” could be felt (Fig. 3A). Restoration was made with glass ionomer cement (Vitremer-3M/ ESPE) (Fig. 3B).

**Figs 4A and B:**
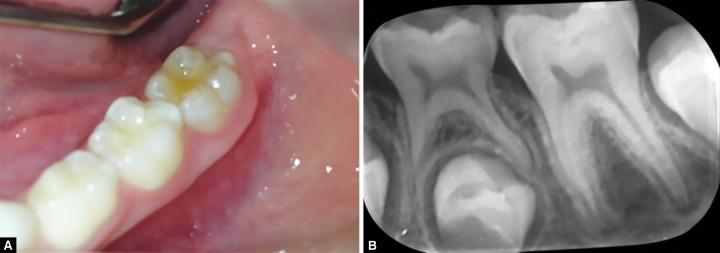
(A) Clinical aspect after 18 months of follow-up since the beginning of clinical approach. (B) Radiograph image of the 18-month follow-up

**Fig. 5:**
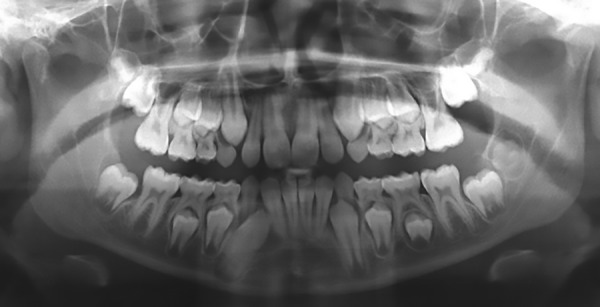
Panoramic radiograph image of the 18-month follow-up

After a period of 6 months, the restoration was made with resin-based composite; the glass ionomer cement was kept as a lining for the restoration. After a follow-up period of 18 months, there were no reports of pain symptomatology and evolution of the rhizogenesis was also observed (Figs 4 and 5).

The project of this clinical case was submitted for evaluation to the Human Research Ethics Committee and approved under number 1.002.654.

## DISCUSSION

The etiology of PECR is unknown. Their pathogenesis is not clear, since cariogenic microorganisms cannot infect the developing teeth in its own bony crypt, which is the case with carious dental lesions. Hence, the term “preemptive caries” is not accepted.^7^ The hypotheses mostly discussed are based on dental development defects that may occur as a result of loss of continuity of the reduced enamel epithelium.^2^

Aspects relating to position seem to play an important role in the etiology of tissue changes. Ectopic position of affected teeth or local pressure of adjacent teeth could trigger a resorption case; in the process, cells responsible for resorption would invade the dentin through “fissures” on the enamel or via amelocemental junction.^1,7^ Seow et al^1^ found a significant association between the ectopic position of teeth and PECR. A loss of integrity in the reduced epithelium of the enamel organ would allow osteoclasts and inflammatory cells to reach the dentin and initiate the resorption process of the tissue.^1^ There was no evidence of ectopic eruption on the reported case, but a delay on the eruption of the affected tooth was noticeable.

An interesting clinical finding of this case is the existence of an “empty dentin” local area corresponding to the radiolucent area. On reaching the radiolucent area, there was no soft tissue to be removed, thereby preventing a histopathological examination of the material. However, there are reports of resorption lacunas containing osteoclasts and macrophages, and a sound layer of dentin between the lesion and the pulp.^3^ Other studies found tissue similar to carious lesions adjacent to the amelodentinal junction, which may extend to different depths of dentin.^4,8^ In some resorption areas, calcified tissue resembling bone tissue replaces the dentin.^2^

The suggested treatment consists of radiographic follow-up until the eruption of the affected tooth, thereafter making an intervention. Many authors therefore, argue that as soon as the diagnosis is made, the affected tooth should be surgically exposed and provisionally restored. After eruption, the material is to be replaced by a permanent one.^2^

On the reported case, it was decided to remove the gin-gival mucosa in order to accelerate the eruption of the tooth affected by PECR. Despite the extensiveness of the radiolu-cent lesion, the decision was taken not to restore the tooth trans-surgically due to difficulty in controlling humidity and bleeding. Clinical and radiographic aspects of the tooth were observed for 6 months, when clinical intervention of the lesion was performed. In the literature, restorative material varies from silver amalgam to resin-based composite.^2,4,7,9^

Although the recommended treatment is surgical intervention to prevent the progression of the lesion to the pulp, it was suggested that shallower lesions could be monitored until eruption to be treated thereafter.^2,7,9,10^ It is essential to emphasize the importance of an early diagnosis for PECR, especially the impacted teeth or teeth experiencing delayed eruption. In such cases, panoramic and interproximal radiographs should be included in routine consultations.
